# New Subregions of the Mouse Entopeduncular Nucleus Defined by the Complementary Immunoreactivities for Substance P and Cannabinoid Type-1 Receptor Combined with Distributions of Different Neuronal Types

**DOI:** 10.1523/ENEURO.0208-22.2022

**Published:** 2022-08-18

**Authors:** Yuta Miyamoto, Takaichi Fukuda

**Affiliations:** Department of Anatomy and Neurobiology, Graduate School of Medical Sciences, Kumamoto University, Kumamoto 860-8556, Japan

**Keywords:** basal ganglia, cannabinoid receptor, electron microscopy, entopeduncular nucleus, parvalbumin, striatum

## Abstract

The entopeduncular nucleus (EPN) and substantia nigra pars reticulata (SNr) constitute the output nuclei of the basal ganglia, but studies on the EPN are limited compared with those on the SNr. Both nuclei receive projections from the striatum with axons containing substance P (SP) and cannabinoid type-1 receptor (CB1R), and immunoreactivities for these substances show complementary patterns in the striatum and SNr. In this study, we revealed a similar complementarity in the mouse EPN, combined it with region-specific neuronal distributions, and defined subregions of the EPN. First, the EPN was divided into two areas, one showing low SP and high CB1R (lSP/hCB1R) immunoreactivities, and the other showing high SP and low CB1R (hSP/lCB1R). The former received inputs from the dorsolateral striatum that are innervated by sensorimotor cortices, whereas the latter received inputs from the medial striatum that are innervated by limbic/association cortices. Then, the lSP/hCB1R area was further divided into the dorsolateral subregion in the rostral EPN and the core subregion in the caudal EPN, the latter characterized by the concentration of parvalbumin-positive neurons targeting the ventral anterior–ventral lateral thalamic nucleus. The hSP/lCB1R area was divided into the ventromedial subregion in the rostral EPN and the shell subregion in the caudal EPN, the former characterized by the concentration of nitric oxide synthase-positive neurons targeting the lateral habenula (LHb). Somatostatin-positive neurons targeting the LHb were located diffusely in three subregions other than the core. These findings illuminate structural organization inside the basal ganglia, suggesting mechanisms for sorting diverse information through parallel loops with differing synaptic modulation by CB1R.

## Significance Statement

The entopeduncular nucleus (EPN) in rodents corresponds to the internal segment of the globus pallidus in primates and is one of the major output nuclei of the basal ganglia. Because studies on the EPN are limited, we investigated the morphological features of the mouse EPN, focusing on the complementary immunoreactivities for substance P (SP) and cannabinoid type-1 receptor (CB1R). The EPN was first divided into two parts, one showing low SP and high CB1R immunoreactivities and the other showing opposite immunoreactivities. Each part was further divided into two subregions based on the distributions of different neuronal types. The present findings clarify the structural organization inside the EPN, suggesting mechanisms for processing diverse information with differing synaptic modulation by CB1R.

## Introduction

The basal ganglia are composed of multiple nuclei located under the cerebral cortex and play an important role in various functions, such as motor regulation, cognition, motivation, and behavioral learning (Alexander and Crutcher, 1990; Graybiel, 2008; Pennartz et al., 2009). The entopeduncular nucleus (EPN) in rodents corresponds to the internal segment of the globus pallidus (GP) in primates, and thus the term “internal globus pallidus” or “medial globus pallidus” is also used in rodents, instead of the EPN. The EPN constitutes, together with the substantia nigra pars reticulata (SNr), the output nuclei of the basal ganglia. The main inputs to both the EPN and SNr originate from the two nuclei, the striatum and subthalamic nucleus (STN). In the striatum, medium-sized spiny neurons of the so-called direct pathway type (dMSNs) project to the EPN and SNr monosynaptically, and they use GABA as a neurotransmitter. In contrast, projection neurons of the STN use glutamate as a neurotransmitter, and they receive GABAergic inputs from the GP, which in turn receives GABAergic innervations from MSNs of the indirect pathway type in the striatum. Thus, information processed in the basal ganglia is converged to the EPN and SNr through direct and indirect pathways. There is an additional pathway termed “hyperdirect,” in which cortical axons directly target the STN (Nambu et al., 2000, 2002). These complex circuits of the basal ganglia are shared by the EPN and SNr, but studies on the EPN, particularly its internal structure, are limited compared with those on the SNr.

The complexity in the basal ganglia circuitry is further increased by the presence of two compartments, striosomes and matrix, in the striatum (Graybiel and Ragsdale, 1978; Crittenden and Graybiel, 2011). Striosomes constitute a labyrinth inside the striatum and can be immunohistochemically identified using numerous molecular markers, such as μ-opioid receptor, substance P (SP), and enkephalin (Graybiel et al., 1981; Gerfen, 1984); these three substances are classical ones that have been used since the discovery of striatal compartmentalization. One essential feature common to most striosomal markers is that they do not label all striosomes equally (Graybiel et al., 1981), but the immunoreactivity changes depending on the position of the striosome inside the striatum (Tajima and Fukuda, 2013). Recently, cannabinoid type-1 receptor (CB1R) was added to the list of striosome markers, and the labeling intensity for CB1R also shows a distinctive regional difference, in both striosomes and the surrounding matrix, with the dorsolateral (DL) striatum showing higher immunoreactivity (Davis et al., 2018). Notably, the gradient in the medial–lateral axis in the striatum also applies to SP immunoreactivity with a pattern opposite to CB1R, and complementarity is further detectable in the SNr (Davis et al., 2018). Therefore, there may be region-specific regulatory mechanisms through CB1R that underlie functional differences among parallel circuits running through the basal ganglia. However, whether similar complementarity in CB1R and SP immunoreactivities can be observed in the EPN remains unknown.

CB1R is a receptor for endocannabinoids (eCBs) and is localized primarily on the plasma membrane of presynaptic neurons in the CNS (Kano et al., 2009). eCB is released from postsynaptic neurons and transmitted retrogradely, and it binds to CB1R localized at presynaptic neurons, thereby suppressing the release of neurotransmitters from presynaptic terminals. This effect is called depolarization-induced suppression of excitation (DSE) or depolarization-induced suppression of inhibition (DSI), depending on the type of synapse involved. CB1R-related synaptic plasticity is found in various brain areas such as the cerebellum and hippocampus (Kreitzer and Regehr, 2001a, b; Ohno-Shosaku et al., 2001; Wilson and Nicoll, 2001). In the basal ganglia, DSI has been reported in the striatum, GP, and SN, taking the form of eCB-mediated short-term depression (Wallmichrath and Szabo, 2002; Engler et al., 2006; Narushima et al., 2006, 2007). Localization of CB1R in the basal ganglia is predominantly in preterminal axons derived from striatal dMSNs (Hohmann and Herkenham, 2000; Mátyás et al., 2006). However, the exact subcellular localization of CB1R has not been demonstrated in the EPN.

In this study, we investigated the labeling patterns of SP and CB1R immunoreactivities in the EPN to determine whether heterogeneity demonstrated in other nuclei of the basal ganglia is also observable in the EPN, and if so, how the internal structure of the EPN can be divided based on apparent heterogeneity in CB1R/SP immunoreactivities that is combined with nonuniform distributions of EPN neurons in each subregion. We also examined subregion-specific connectivity between the striatum and EPN, which resulted in strengthening the newly proposed subregions of the EPN. The present findings will update knowledge about the internal structure of the EPN and provide clues to consider how diverse information is integrated into the basal ganglia circuitry.

## Materials and Methods

### Fixation and tissue preparation

All experiments in this study were performed according to the *Guide for Care and Use of Laboratory Animals* (National Institutes of Health Publications No. 80–23, revised 1996), and all protocols were approved by the Institutional Animal Care and Use Committees at our university. All efforts were made to minimize the number of animals used and their suffering.

Thirty-three male C57BL/6J mice (weight, 20–26 g; age, 7–8 weeks) were deeply anesthetized by the inhalation of isoflurane. Thirty of the animals were perfused via the ascending aorta with 0.01 m PBS, pH 7.4, followed by 50 ml of 4% paraformaldehyde (PFA) in 0.1 m phosphate buffer (PB), pH 7.4, at room temperature. The remaining three mice were perfused using the same procedure, but 4% PFA was replaced by 4% PFA and 0.1% glutaraldehyde in 0.1 m PB at pH 7.2 for electron microscopic analysis. Two hours later, the brain was removed from the skull and stored overnight in PFA at 4°C. The fixative was replaced with PBS containing 0.1% sodium azide the next day.

### Injection of anterograde tracers

The anterograde tracer used was phaseolus vulgaris-leucoagglutinin (phal, 2.5% in 10 mm PB, pH 8.0; Vector Laboratories). Phal was injected stereotaxically into four parts of the striatum: two different DL positions [anteroposterior (AP), −0.11 mm; mediolateral (ML), 2.85 mm; dorsoventral (DV), 2.25 mm; and AP, 0.37 mm; ML, 2.5 mm; DV, 1.75 mm; *n* = 3 animals each); a dorsomedial (DM) position (AP, 0.14 mm; ML, 1.25; DV, 2.13 mm; *n* = 3 animals); and ventromedial (VM) position (AP, 0.85 mm; ML, 1.0 mm; DV, 2.87 mm; *n* = 3 animals). Mice were anesthetized by inhalation of 0.5–2.0% isoflurane and mounted in a stereotaxic frame (model SR-5 M-HT, Narishige Scientific Instrument Lab). Then, a burr hole was drilled in the appropriate position of the skull, and a glass microelectrode (outside tip diameter, 40 μm) containing tracer solutions was stereotaxically inserted into the brain. Phal was injected iontophoretically into targeted sites by passing a positive-pulsed 5–7 μA duty cycle (2 s on/2 s off) for 5 min. After surgery, the skin incision was closed and a topical analgesic (2% lidocaine gel; Fujisawa) was applied. Mice were housed singly in small compartments that were temperature controlled (20°C) and light controlled (12 h light/dark cycle). After a survival period of 7 d, the mice were perfusion fixed with 4% PFA in 0.1 m PB, as described above.

### Immunohistochemistry and confocal laser-scanning microscopy

Serial 40-μm-thick coronal sections were cut using a vibrating microtome (model TTK-3000, Dosaka) from brain blocks containing the entire striatum and EPN. After cryoprotection in 25% sucrose in PBS, sections placed on aluminum foil were rapidly frozen in the vapor of liquid N_2_, rapidly thawed in 25% sucrose in PBS, and then processed for triple-fluorescent immunohistochemistry. The primary and secondary antibodies used in this study are listed in Tables 1 and 2, respectively. Briefly, sections were incubated in 5% normal donkey serum (Jackson ImmunoResearch) and 0.3% Triton X-100 in PBS overnight, followed by a mixture of rat anti-SP (1:500; Millipore), rabbit anti-CB1R (1:500; Frontier Institute), and mouse anti-calbindin D-28 k (CALB; 1:5000; Swant) antibodies for 5–7 d at 20°C, with biotinylated donkey anti-rat IgG (1:250; Jackson ImmunoResearch) overnight, and with a mixture of rhodamine red-conjugated donkey anti-rabbit IgG (1:250; Jackson ImmunoResearch), streptavidin-Alexa Fluor 647 (1:250; Jackson ImmunoResearch), and Alexa Fluor 488-conjugated donkey anti-mouse IgG (1:250; Jackson ImmunoResearch) overnight. The long incubation period with primary antibodies was essential to improve permeation of the antibodies into the deep portions of the 40-μm-thick sections to obtain confocal images of consistent and sufficient quality throughout the depth of the sections. The second set of triple immunostaining was performed using a mixture of goat anti-CB1R (1:500; Frontier Institute), rabbit anti-glutamic acid decarboxylase (GAD; 1:5000; Sigma-Aldrich), and guinea pig anti-vesicular glutamate transporter type 2 (vGluT2; 1:1000; Frontier Institute) antibodies, followed by the same procedures as described for the first set of triple immunostaining. Several other sets of immunostaining were performed by combining the primary and secondary antibodies listed in Tables 1 and 2 to investigate the distribution of the following four types of EPN neurons: parvalbumin (PV)-, nitric oxide synthase (NOS)-, somatostatin (SOM)-, and choline acetyltransferase (ChAT)-containing neurons. Immunolabeled sections were mounted in Vectashield (Vector Laboratories) and examined using a confocal laser-scanning light microscope (model C2, Nikon), equipped with three single laser beams, 488, 543, and 633 nm in wavelength, and a filter set of BA 515/30, BA 590/50, and low pass 650. Control sections were prepared by omitting primary antibodies and mismatching secondary antibodies. Both sets of controls provided only weak, nonspecific staining.

**Table 1:** Primary antibodies and dilutions used in the study

Antibody	Host species	Dilution	Source	Catalog no.	RRID
CALB D-28 k	Mouse	1:5000	Swant	300	AB_10000347
CB1R	Rabbit	1:1000	Frontier institute	CB1-Rb-Af380	AB_2571591
CB1R	Goat	1:500	Frontier institute	CB1-Go-Af450	AB_2571530
CB1R	Guinea pig	1:500	Frontier institute	CB1-GP-Af530	AB_2571593
SP	Rat	1:500	Millipore	MAB356	AB_94639
GAD65/67	Rabbit	1:5000	Sigma-Aldrich	G5163	AB_477019
vGluT2	Guinea pig	1:1000	Frontier Institute	VGluT2-GP-AF810	AB_2341096
SOM	Rat	1:250	Millipore	MAB354	AB_2255365
SOM	Rabbit	1:1000	Gene Tex	GTX133119	AB_2814698
PV	Mouse	1:5000	Swant	235	AB_10000343
NOS	Sheep	1:10 000	Gift from Dr. Emson PC		AB_2895154
ChAT	Goat	1:1000	Millipore	AB144P	AB_2079751
Phal	Rabbit	1:2500	Vector Laboratories	AS-2300	AB_2313686

**Table 2:** Secondary antibodies and dilutions used in the study

Antibody and fluorochrome	Conjugated fluorophore	Dilution	Source	Code no.	RRID
Donkey anti-rat IgG	Biotin	1:250	Jackson ImmunoResearch	712–065-153	AB_2315779
Donkey anti-guinea pig IgG	Biotin	1:250	Jackson ImmunoResearch	706–065-148	AB_2340451
Donkey anti-mouse IgG	Alexa Fluor 488	1:250	Jackson ImmunoResearch	715–545-151	AB_2341099
Donkey anti-rabbit IgG	Alexa Fluor 488	1:250	Thermo Fisher Scientific	A21206	AB_2535792
Donkey anti-rabbit IgG	Rhodamine red	1:250	Jackson ImmunoResearch	711–295-152	AB_2340613
Donkey anti-goat IgG	Rhodamine red	1:500	Jackson ImmunoResearch	705–295-147	AB_2340423
Streptavidin	Alexa Fluor 647	1:250	Jackson ImmunoResearch	016–600-084	AB_2341101

Images for confocal laser-scanning light microscopy (CLSM) were obtained using 4× [Plan Apo, numerical aperture (NA) 0.2; Nikon], 10× (Plan Fluor, NA 0.3; Nikon), 20× (Plan Fluor, NA 0.5; Nikon), and 60× (Plan Apo VC, NA 1.4, Nikon) objectives. The 4× and 10× objectives were used to visualize the entire striatum and EPN within a single frame of the CLSM, whereas the 20× and 60× objectives were used to identify and analyze EPN neurons and axons with sufficient resolution. The size of each frame was 1024 × 1024 pixels, and images of optical slices were acquired from the section surface to the bottom at the preset optimal step size and were stored as stacked files for each frame using the three single laser beams alternately at each *z*-position of the stage to collect images of different fluorescence signals. The intensity of the signal in each pixel was recorded at 8 bits for each channel.

### Electron microscopic observations of CB1R immunoreactivity

Sections (40 μm) from animals fixed with 4% PFA and 0.1% glutaraldehyde in 0.1 m PB were processed for immunoelectron microscopy. After rapid freezing-thawing as above, sections were incubated at 4°C with guinea pig primary antibody against CB1R (1:500) for 7 d and with biotinylated donkey anti-guinea pig IgG (1:250) overnight and then treated with a standard ABC kit (Vector Laboratories) for 2 h at room temperature. Detergent was omitted from all solutions. After color development with diaminobenzidine (DAB), sections were postfixed with 1% OsO_4_ in 0.1 m PB for 1.5 h on ice, stained en bloc with 1.5% uranyl acetate, dehydrated, and embedded in Araldite. Sections 65 nm in thickness were cut from the superficial part of the re-embedded specimens, lightly stained with uranyl acetate and lead citrate, and examined under a transmission electron microscope (model HT-7700, Hitachi).

### Analysis

To investigate the gray values of SP and CB1R signals in CLSM (see Fig. 3), coronal section images including the EPN were acquired using a 10× objective. The number of sections used in the analysis was four per animal (two sections at rostral positions, −1.08 and −1.24 mm from bregma; two sections at caudal positions, −1.40 and −1.56 mm from bregma), and the data were collected from three animals. The extent of EPN was defined as the region showing SP immunoreactivity within the internal capsule because axon terminals of striatal dMSNs contain SP. Gray values of fluorescent signals in CLSM images (no signal = 0, maximum level = 255) were measured for SP and CB1R labeling inside the EPN by the following procedure (see Fig. 3*a*) using the public domain program ImageJ (version 1.47), as follows: (1) original images were acquired by CLSM; (2) pseudocolor images were split into images in single channels; (3) a grid of 127 × 127 μm in frame size was applied to each CLSM image; (4) frames used for measurements (see Fig. 3*a*, green frames) were selected from the frames covering the EPN according to the systematic random sampling rule [i.e., the first frame was selected at random from one of three frames located at the top left of the squares covering the EPN (e.g., no. 2 selected from nos. 1–3), then one of three frames was selected successively from top left to bottom right (nos. 5, 8, 11, 14)]; and (5) pixelwise gray values of SP and CB1R signals were measured and averaged in each frame. The areas occupied by the internal capsule were traced in each frame and excluded in measurements of gray values.

For quantitative analysis of the number and numerical density of EPN neurons in each subregion (see Fig. 8), every other section, which was 80 μm apart from each other, was selected from serial sections covering the entire EPN; four sections in the rostral half and four sections in the caudal half were selected for analysis as a whole. Data were collected from three animals. CLSM images were acquired with a 10× objective to determine the boundary of each subregion and with a 20× objective to count cells inside each subregion, using the computer-assisted neuron tracing system Neurolucida (MBF Bioscience). The boundary of the EPN was determined using SP immunostaining, and that of the low SP and high CB1R (lSP/hCB1R) region was determined using CB1R immunostaining. The area included inside the boundary of the EPN but outside the lSP/hCB1R boundary was defined as the high SP and low CB1R (hSP/lCB1R) region. Each boundary was traced using Neurolucida. To confirm the accuracy of tracings, the mean gray value of CB1R was measured in each subregion in the above eight sections (*n* = 24 sections from three animals), before cell counting. Measurements in each animal were normalized so that the highest value in 16 datasets (8 datasets from lSP/hCB1R regions in 8 sections and 8 datasets from hSP/lCB1R regions in the same sections) was set to 1 (see Fig. 9).

The number of somata located inside each subregion was counted at eight different positions along the rostrocaudal axis, and the data from three animals were averaged at each position (see Fig. 8*a*,*b*). According to the principle of the unbiased stereological disector method (Sterio, 1984), the somata that were exposed to the top surface of each section were identified by examining serial optical slices in CLSM and were excluded from the quantification. The number of cells was divided by the area of each subregion and further by the section thickness to yield cell density per unit volume at eight positions (see Fig. 8*c*,*d*). In the quantitative analyses seen in Figures 10 and 11, the EPN was divided into rostral halves (rEPN) and caudal halves (cEPN) at the rostrocaudal level between 1.28 and 1.36 mm caudal to bregma. The lSP/hCB1R and hSP/lCB1R regions in the rostral half were termed the DL and VM subregions, respectively, while the lSP/hCB1R and hSP/lCB1R regions in the caudal half were termed the core and shell. The cell densities of the four types of neurons in each subregion were obtained from four sections located at different positions along the rostrocaudal axis (for DL and VM, −1.04, −1.12, −1.2, and −1.28 mm from bregma; for core and shell, −1.36, −1.44, −1.52, and −1.6 mm from bregma) and then averaged (*n* = 3 animals).

### Statistics

All quantitative data were expressed as the mean ± standard deviation (SD) and were analyzed by the Mann–Whitney *U* test and Tukey–Kramer test in the public program R, with *p* < 0.05 considered statistically significant.

## Results

### Contrasting immunoreactivities for SP and CB1R in the striatum and EPN

**Figure 1.**
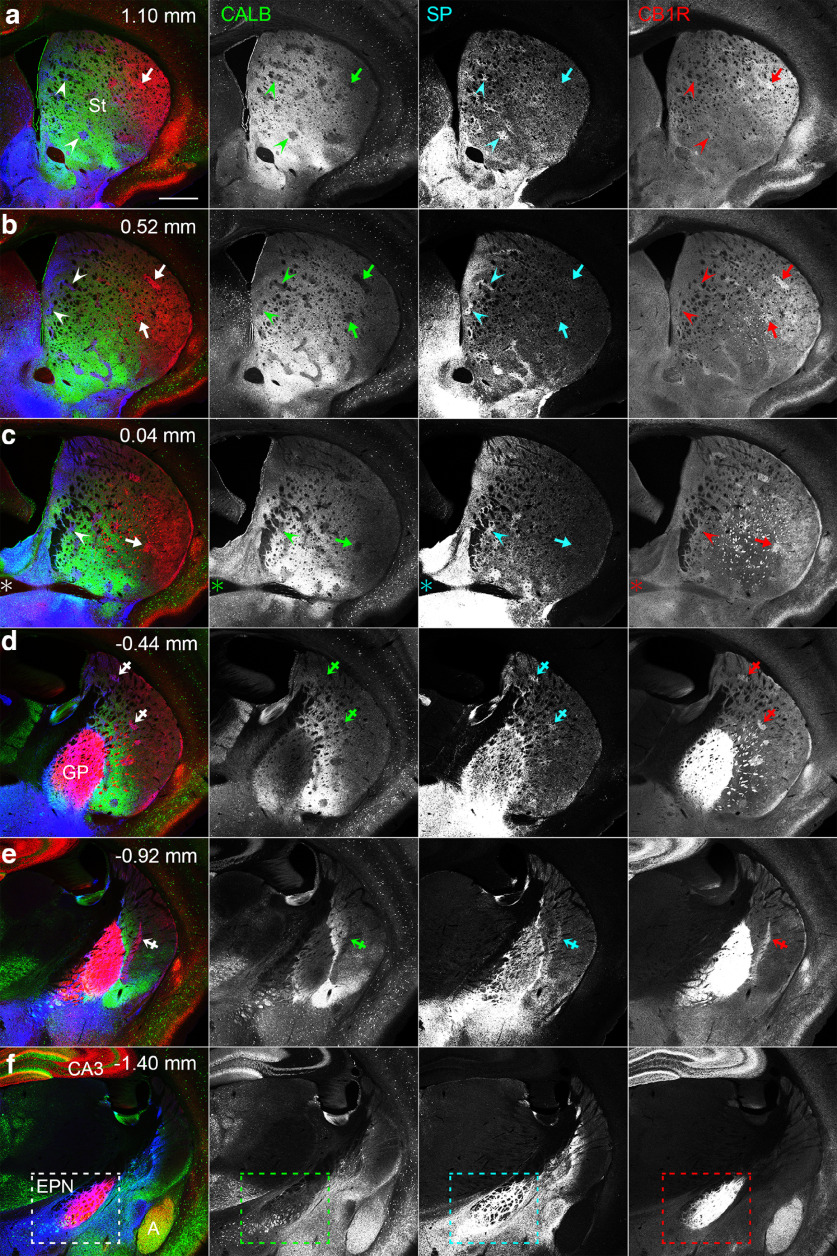
CLSM images of triple-labeled immunohistochemistry for CALB, SP, and CB1R in coronal sections containing the basal ganglia. ***a***–***f***, Pseudocolor images consist of CALB (green), SP (blue), and CB1R (red) immunoreactivities, which are shown separately in the right panel. Arrows, arrowheads, and crossed arrows indicate lSP/hCB1R-immunoreactive, hSP/lCB1R-immunoreactive, and hSP/hCB1R-immunoreactive striosomes, respectively. In CALB immunostaining, striosomes are shown as unstained islands. The number at the top right of each image represents the distance from bregma. The rectangle in ***f*** indicates the EPN, which is characterized by intense CB1R and SP labeling. The asterisk in ***c*** indicates the position where the anterior commissure crosses the midline. A, Amygdala; CA3, CA3 region of the hippocampus; St, striatum. Scale bar, 600 μm.

Serial coronal sections containing the striatum, GP, and EPN were processed for triple immunohistochemistry using antibodies against CALB, SP, and CB1R (Fig. 1). In the striatum, CALB immunoreactivity was used to detect the positions of striosomes because CALB is a general marker of the matrix and surrounds CALB-poor striosomes (Gerfen et al., 1985). SP and CB1R immunoreactivities were observed in all of the striatum, GP, and EPN (Fig. 1). In the striatum, both immunoreactivities showed position-dependent heterogeneity that was consistent with previous observations (Davis et al., 2018). The heterogeneity in CB1R labeling in the striatum was evident in the colored images in Figure 1, in which CB1R (shown in red) was more intense in the lateral part of the striatum (Fig. 1*a–d*). Close observations revealed that the intense CB1R immunoreactivity in the lateral part was observed in both major compartments of the striatum, striosomes (Fig. 1, arrows), and the surrounding matrix. Regarding SP immunoreactivity, the heterogeneity was found in the immunoreactivity inside striosomes with a pattern in contrast to that in CB1R immunoreactivity. Namely, medially located striosomes (Fig. 1, arrowheads) showed higher SP immunoreactivity than laterally located striosomes. Thus, striosomes showing hSP/lCB1R immunoreactivities were predominantly located in the medial part of the striatum, whereas lSP/hCB1R immunoreactivities were observed in striosomes and matrix located in the lateral part of the striatum (Fig. 1*a–c*). An exceptional pattern was found, however, in the caudal striatum (Fig. 1*d*,*e*). In the region posterior to the landmark along the rostrocaudal axis, where the anterior commissure crossed the midline (∼0 mm from bregma), striosomes showed relatively high immunoreactivities for both SP and CB1R (hSP/hCB1R; Fig. 1*d*,*e*, crossed arrows).

**Figure 2.**
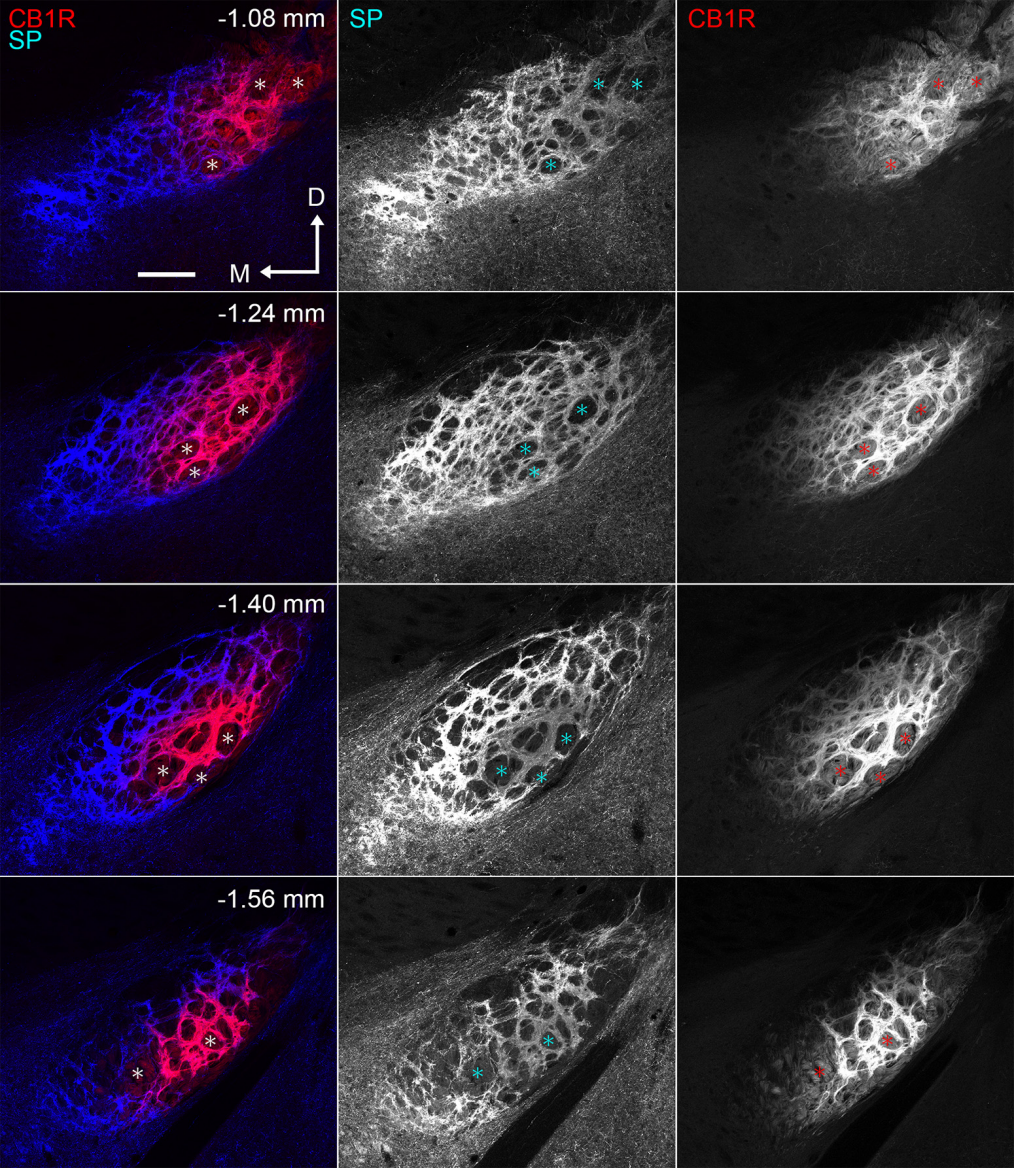
Double immunohistochemistry for SP and CB1R in the EPN at four different positions along the rostrocaudal axis. The number in each image represents the distance from bregma. CLSM settings are optimized to acquire images of the internal structure of the EPN. Asterisks show fibers passing through the white matter. Immunoreactivities for SP and CB1R show complementary patterns at all rostrocaudal levels. M, Medial; D, dorsal. Scale bar, 200 μm.

Immunoreactivities for SP and CB1R in the GP and EPN were much higher than those in the striatum and some other regions outside the basal ganglia, such as CB1R in the hippocampus and amygdala (Fig. 1*d–f*). Therefore, when CLSM settings were adjusted to SP and CB1R signal levels in the striatum, signals in the GP and EPN became saturated and appeared homogeneously intense (Fig. 1*d–f*). In the present study, we intended to acquire CLSM images under the condition that was optimized to examine the internal structure of the EPN. Under this condition, the laser power setting did not cause halation in either SP or CB1R labeling in the EPN (Fig. 2). As a result, the immunoreactivities for SP and CB1R inside the EPN exhibited patterns complementary to each other. In the rostral EPN (−1.04 to −1.32 mm from bregma), SP immunoreactivity was stronger in the VM part and tended to become weaker toward the DL part. In contrast, CB1R immunoreactivity was lower or almost absent in the VM part and became stronger in the DL part. In the cEPN (−1.32 to −1.68 mm from bregma), intense CB1R immunoreactivity was observed in the central part of the EPN. In contrast, intense SP immunoreactivity was distributed to surround the central part where intense CB1R immunoreactivity was observed. We used three different antibodies for CB1R immunostaining (Table 1), and all of them provided the consistent labeling patterns. In the following analyses, the position at −1.32 mm from bregma where SP/CB1R labeling pattern changes was set to the border between the rostral and caudal EPN.

**Figure 3.**
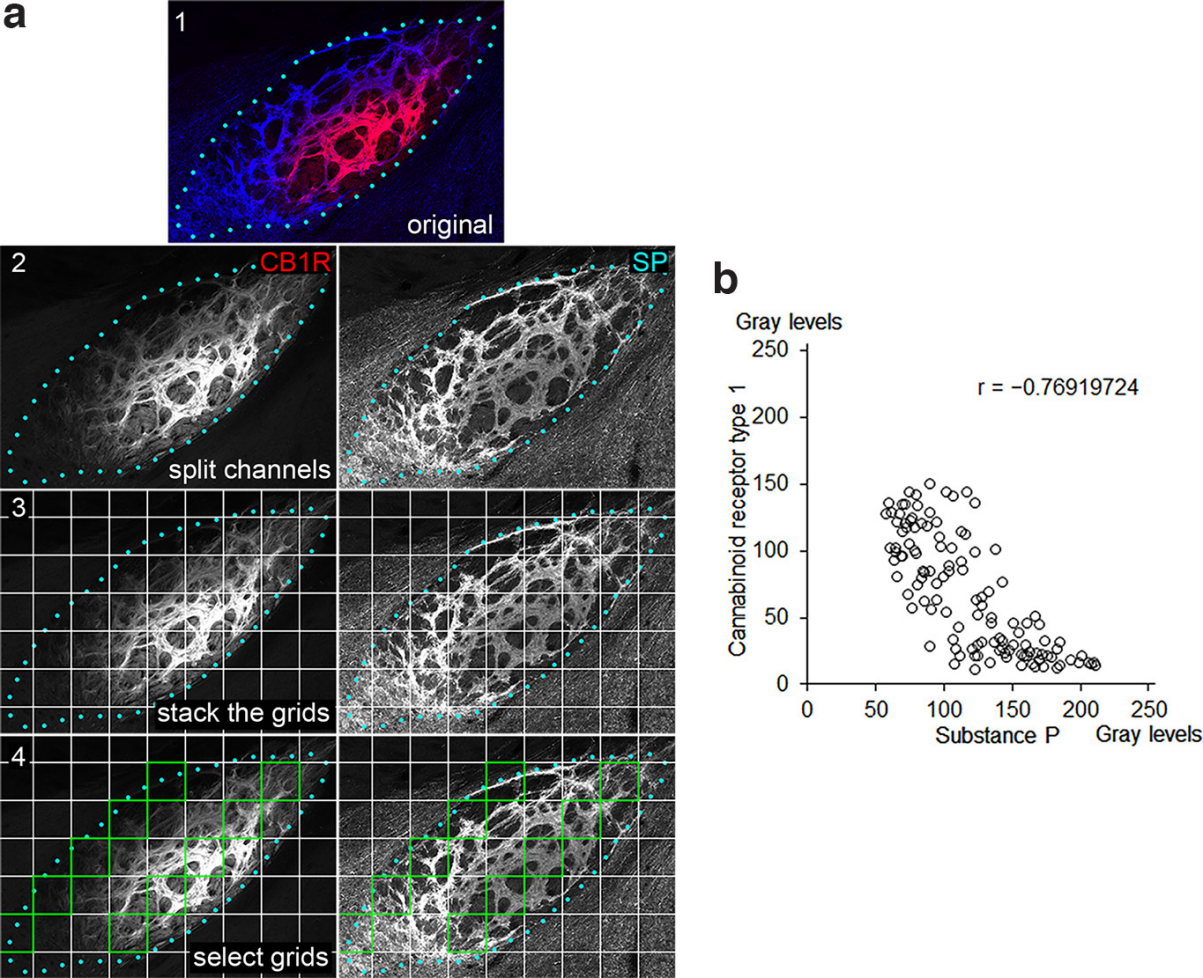
Analysis of contrasting immunoreactivities for SP and CB1R in the EPN. ***a***, The procedures to measure the gray values of immunoreactivities for SP and CB1R. The dual-label image (1) is split into two singe-channel images (2), which are overlaid with a grid (3). The frames to be analyzed are selected according to the systematic random sampling method and are colored green (***a***, 4). Blue dots represent the boundary of the EPN determined by SP immunoreactivity. ***b***, Correlation diagram showing the intensities of CB1R and SP immunoreactivities measured within the green frames. Each dot corresponds to the averaged gray value inside each frame, in which areas occupied by the white matter are excluded in measurements (*n* = 40 frames; 4 sections in each animal; 3 animals were used).

The complementary patterns for SP and CB1R immunoreactivities in the EPN were quantitatively analyzed by measuring gray values of fluorescence signals in CLSM images (Fig. 3). A grid with a frame size of 127 × 127 μm was placed on the CLSM images, and pixelwise gray values were measured and averaged inside each frame separately for SP and CB1R. The obtained measurements were plotted in a scatter diagram (Fig. 3*b*). The tendency of contrasting immunoreactivities for SP and CB1R was confirmed statistically using R software to calculate the correlation coefficient (*r* = –0.76919724, *p* = 1.06 × 10^−24^).

### Subcellular localization of CB1R in the EPN

**Figure 4.**
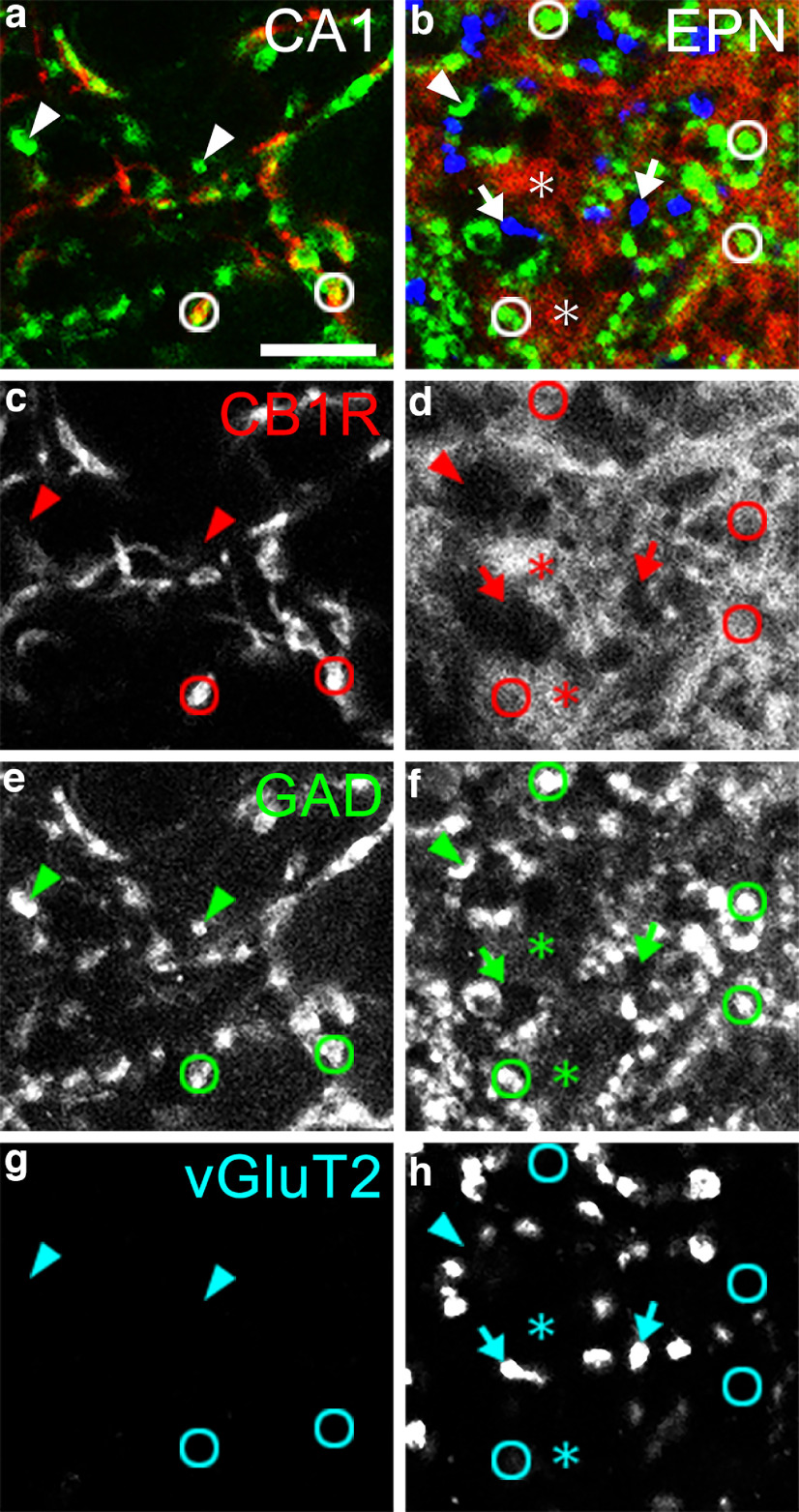
***a–h***, CLSM images of triple-labeled immunohistochemistry for CB1R, GAD, and vGluT2 in the CA1 region of the hippocampus (***a***, ***c***, ***e***, ***g***) and lSP/hCB1R-immunoreactive area of the EPN (***b***, ***d***, ***f***, ***h***). The pseudocolor images (***a***, ***b***) consist of CB1R (red), GAD (green), and vGluT2 (blue) immunoreactivities, each displayed in the bottom panel (***c–h***). Circles, arrows, and arrowheads show CB1R/GAD-double positive, vGluT2-only positive, and GAD-only positive boutons, respectively. Asterisks represent the regions showing intense CB1R immunoreactivity outside boutons. Scale bars, 10 μm.

Coronal sections containing the hippocampus and EPN were processed for triple immunohistochemistry using antibodies against CB1R, GAD, and vGluT2 (Fig. 4). The pyramidal cell layer of the CA1 region of the hippocampus was selected to demonstrate the quality of immunostaining for CB1R because CB1R in this region has been well documented (Egertová and Elphick, 2000; Kano et al., 2009). In general, the subcellular localization of CB1R in the CNS has been reported to be in axon terminals and preterminal axons (Mátyás et al., 2006; Uchigashima et al., 2007; Kano et al., 2009). In the present analysis, CB1R immunoreactivity in the CA1 regions was observed in boutons and axons, and the labeled boutons colocalized GAD (Fig. 4*a*,*c*,*e*, circles), although not all GAD-positive boutons showed CB1R immunoreactivity (Fig. 4*a*,*c*,*e*, arrowheads). These results were consistent with previous findings at the light microscope level (Egertová and Elphick, 2000; Kano et al., 2009). In the EPN, CB1R immunoreactivity was detected as a diffuse meshwork pattern rather than discrete boutons, as in the hippocampus (Fig. 4*d*). However, close observations revealed that weak CB1R immunoreactivity overlapped with GAD-positive boutons (Fig. 4*d*,*f*, circles). This was contrasted with no CB1R staining in vGluT2-positive boutons (Fig. 4*b*,*d*,*h*, arrows), except for very rare cases in which vGluT2-positive boutons were faintly labeled for CB1R. Some GAD-positive boutons did not show CB1R immunoreactivity as in the hippocampus (Fig. 4*d*,*f*, arrowheads). Diffuse, intense CB1R labeling that did not overlap with GAD-positive boutons (Fig. 4*b*,*d*,*f*, asterisks) was thought to represent immunoreactivity in accumulated axons, which we confirmed by electron microscopy (EM; see below).

**Figure 5.**
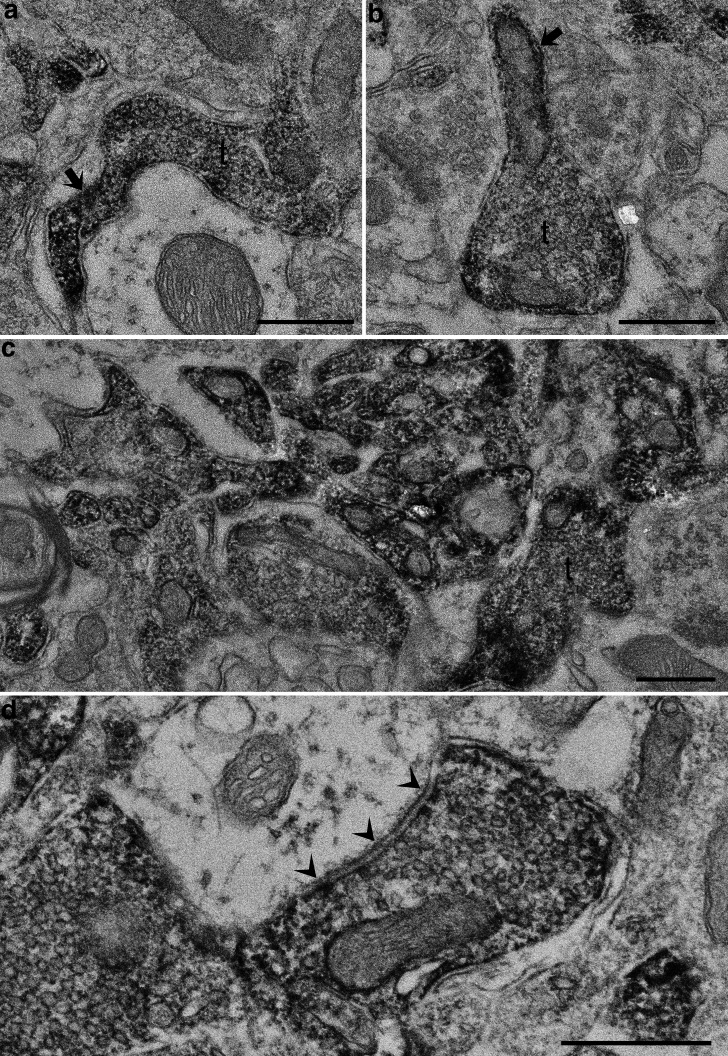
***a–d***, Electron micrographs illustrating the immunohistochemical localization of CB1R in the EPN. CB1R-immunoreactive elements labeled by DAB are located in preterminal axons (***a***, ***b***, arrows) and axon terminals (t). The accumulation of labeled axons and axon terminals shown in ***c*** is consistent with diffuse, intense labeling observed in CLSM. Arrowheads in ***d*** indicate a symmetrical synapse. Scale bars, 0.5 μm.

The more accurate localization of CB1R in the EPN was examined using electron microscopy (Fig. 5). CB1R immunoreactivity was observed in preterminal axons and axon terminals forming synapses of symmetrical type. This result was consistent with previous observations in other basal ganglia nuclei, such as the GP and SNr (Mátyás et al., 2006; Uchigashima et al., 2007). The accumulation of labeled axons (Fig. 5*c*) was thought to correspond to areas showing diffuse CB1R labeling in CLSM images (Fig. 4, asterisks). Notably, DAB reaction products inside axon terminals in the EPN were located mainly at the peripheral part near the plasma membrane rather than in the central part where synaptic vesicles accumulated. This pattern is consistent with the localization of CB1R molecules in the plasma membrane (Mátyás et al., 2006; Uchigashima et al., 2007) and further explains the rather weak immunofluorescence signals for CB1R in GAD-positive boutons, where the localization of GAD, the GABA-synthetic enzyme, is associated with numerous synaptic vesicles accumulating in axon terminals and causing discrete labeling patterns for GAD in boutons.

### Distribution of four types of neurons in the EPN

**Figure 6.**
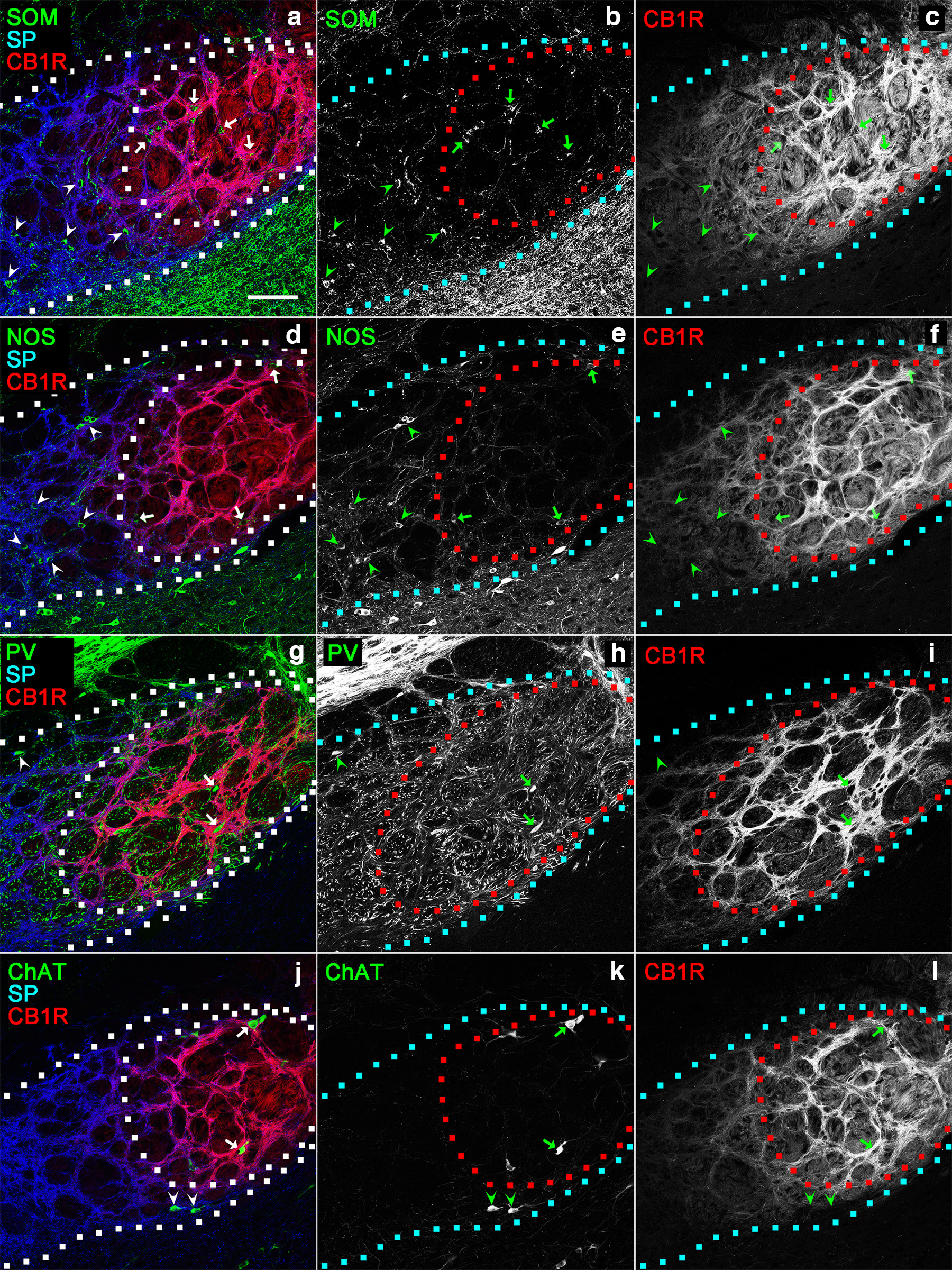
Distributions of four types of neurons in the rEPN. In the pseudocolor images, immunostaining for SOM (***a***), NOS (***d***), PV (***g***), or ChAT (***j***) is shown in green, and immunostaining for SP and CB1R is shown in blue and red, respectively. ***b***, ***c***, ***e***, ***f***, ***h***, ***i***, ***k***, ***l***, The green and red images are shown separately. The boundary of the entire EPN and that of the lSP/hCB1R-immunoreactive area are drawn by dashed lines. Arrowheads and arrows indicate neurons localized within hSP/lCB1R and lSP/hCB1R areas. Scale bar, 100 μm.

**Figure 7.**
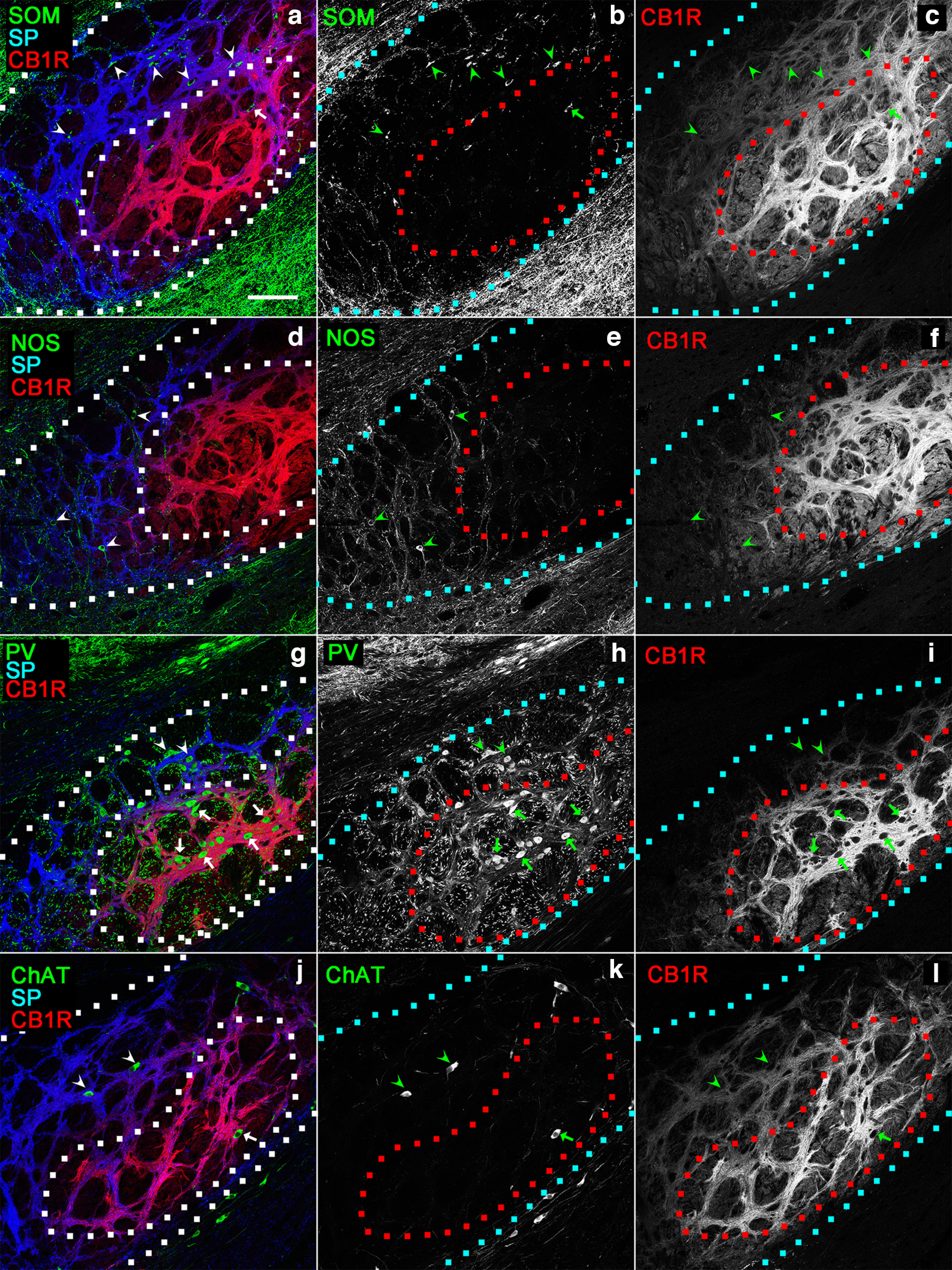
Distributions of the four types of neurons in the cEPN. The compositions of pseudocolor images, symbols, and dashed lines are as in Figure 6. Scale bar, 100 μm.

According to previous quantitative studies using stereology, SOM-, NOS-, PV-, and ChAT-containing neurons as a whole comprise nearly the entire population of EPN neurons (Miyamoto and Fukuda, 2015, 2021). Moreover, none of antibodies against calbindin, calretinin, vasoactive intestinal peptide, and neuropeptide Y labeled neurons inside the EPN (Miyamoto and Fukuda, 2015). Thus, the distribution of SOM-, NOS-, PV-, and ChAT-containing neurons was investigated in areas with hSP/lCB1R and lSP/hCB1R immunoreactivities in the EPN. Serial coronal sections containing the entire EPN were processed for triple immunohistochemistry using antibodies against SP, CB1R, and an additional molecule of either SOM, NOS, or PV (Figs. 6, 7). SOM-containing neurons in the rEPN were scattered diffusely in both areas, showing hSP/lCB1R and lSP/hCB1R immunoreactivities (Fig. 6*a–c*). In contrast, SOM-containing neurons in the cEPN were distributed in the area with hSP/lCB1R immunoreactivity (Fig. 7*a–c*). A previous study showed that the majority of NOS-containing neurons was distributed in the VM part of the rEPN (Miyamoto and Fukuda, 2021). The present analysis revealed that NOS-positive neurons in the rEPN were localized predominantly in the area with hSP/lCB1R immunoreactivity, corresponding to the VM part of the rEPN (Fig. 6*d–f*), although a small number of NOS-containing neurons was also observed in the lSP/hCB1R area of the rEPN. In the cEPN, a small number of NOS-containing neurons was identified in the area with hSP/lCB1R immunoreactivity, whereas NOS neurons were rarely observed in the lSP/hCB1R area (Fig. 7*d–f*). The localization of PV-containing neurons is reportedly concentrated in the caudal part of the EPN (Miyamoto and Fukuda, 2015, 2021). In this study, we demonstrated that most PV-containing neurons accumulated in the area with lSP/hCB1R immunoreactivity in the cEPN (Fig. 7*g–i*). A very rare occasion of PV-containing neurons in the rEPN also tended to be distributed in the area with lSP/hCB1R immunoreactivity (Fig. 6*g–i*). ChAT-containing neurons are the smallest subpopulation in the EPN and are mainly distributed in the lateral part of the EPN and at the boundary with the GP (Miyamoto and Fukuda, 2015). They were observed within the EPN independent of the immunoreactivity for SP and CB1R (Figs. 6*j–l*, 7*j–l*).

**Figure 8.**
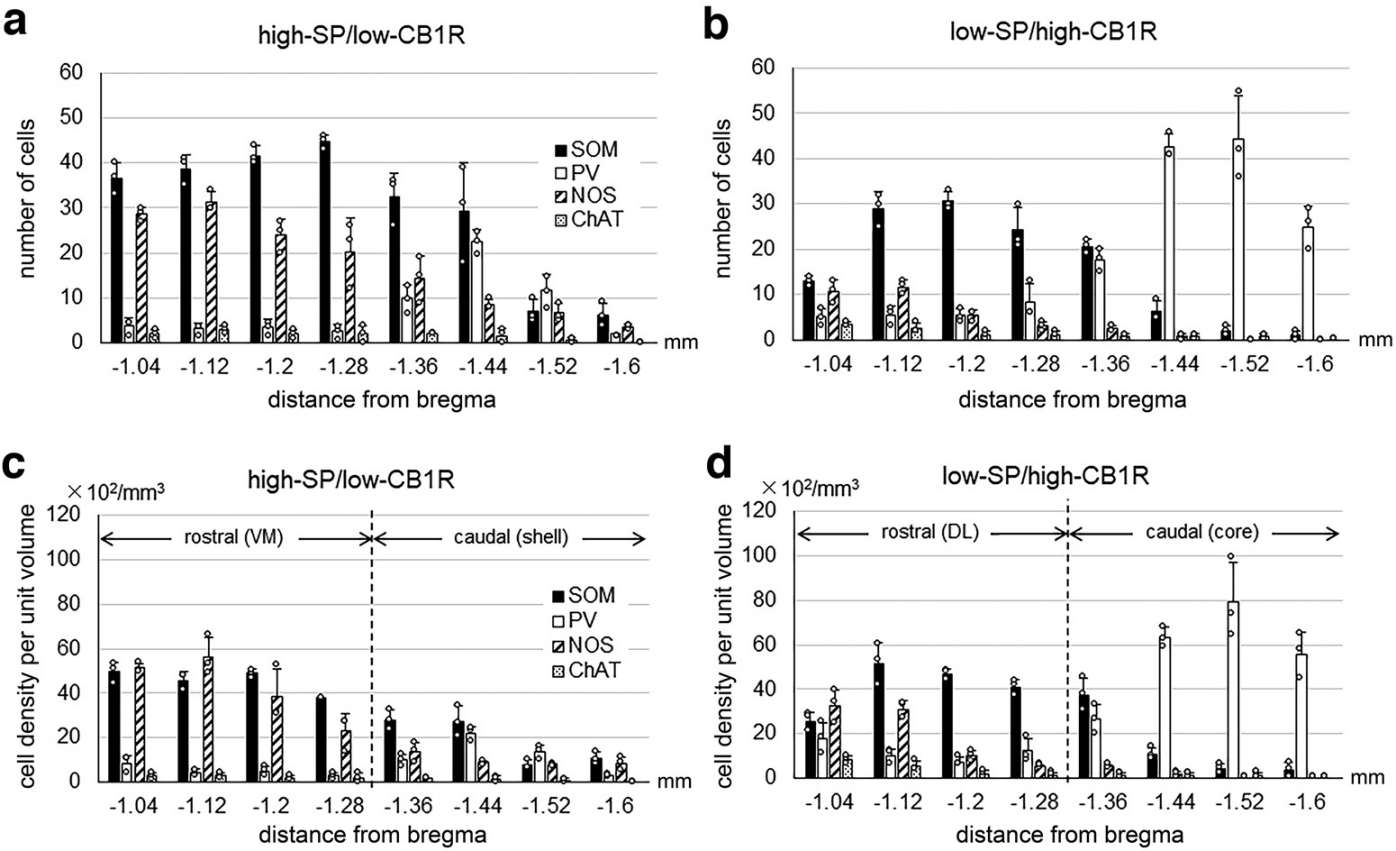
Distributions of four types of neurons along the rostrocaudal axis of the EPN. The abscissa shows the position relative to bregma (in mm). ***a–d***, The ordinate shows the number of cells in a single section (***a***, ***b***) and the cell density per unit volume (μm^3^; ***c***, ***d***). The vertical dashed line drawn between −1.28 and −1.36 mm from bregma indicates the position along the rostrocaudal axis, where SP/CB1R labeling changes from DL/VM pattern to core/shell pattern. Data at each position were collected from three animals.

**Figure 9.**
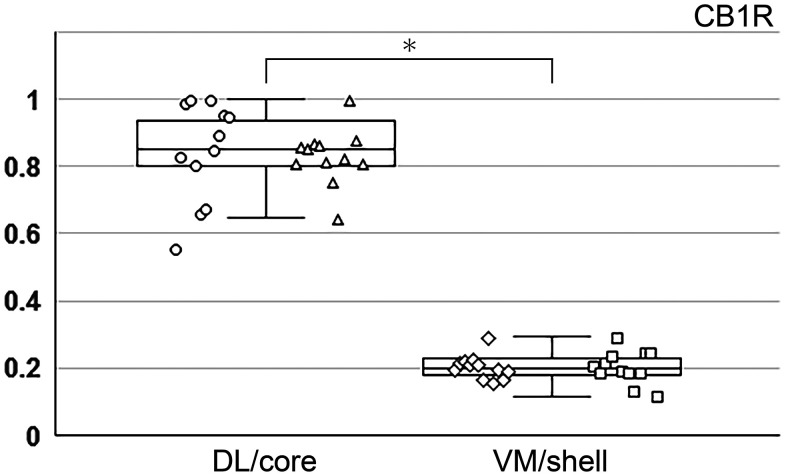
Comparison of CB1R gray values between hSP/lCB1R-immunoreactive and lSP/hCB1R-immunoreactive areas, the boundaries of which were predetermined before cell counting. Circles, triangles, trapezoids, and squares show the mean gray values in the DL, core, VM, and shell subregions, respectively. Measurements in the DL and VM were performed at 4 different positions (−1.04, −1.12, −1.2, −1.28 mm from bregma) in the rEPN, whereas those in the core and shell were performed at four different positions (−1.36, −1.44, −1.52, −1.6 mm from bregma) in the cEPN. Data were collected from 3 animals; thus 12 datasets acquired from 4 different positions are shown by the same symbol. In each animal, the dataset of gray values at 8 positions (4 rostral positions and 4 caudal positions) was normalized so that the highest value among 8 positions was set to 1. Datasets of gray values for lSP/hCB1R areas (DL and core, *n* = 24) and hSP/lCB1R areas (VM and shell, *n* = 24) were statistically compared using the Mann–Whitney *U* test. *p < 1.0 × 10^−7^.

The distribution of EPN neurons in the areas characterized by differential SP and CB1R immunoreactivities was further analyzed quantitatively using the disector method (Sterio, 1984). The localizations of the four types of EPN neurons were analyzed separately for the areas showing hSP/lCB1R and lSP/hCB1R immunoreactivities (Fig. 8). In this analysis, the boundaries of these two areas were predetermined using low-magnification images (10×) of CB1R immunoreactivity, not observing labeled neurons. To confirm the accuracy of manual tracings of the boundaries, the mean gray value for CB1R was measured and compared between the two areas (Fig. 9). The graph clearly indicates the difference in CB1R labeling intensity between the two areas with a statistically significant difference (*n* = 24 for each of hSP/lCB1R and lSP/hCB1R area; Mann–Whitney *U* test, *p* < 1.0 × 10^−7^).

The result of the quantitative analysis of the neuronal distribution was shown by both the number of cells in a single section (Fig. 8*a*,*b*) and the cell density per unit volume (Fig. 8*c*,*d*). SOM-containing neurons were predominantly distributed in the rEPN in both the hSP/lCB1R (Fig. 8*a*,*c*) and lSP/hCB1R (Fig. 8*b*,*d*) areas. NOS-containing neurons were also distributed abundantly in the rEPN, especially in the area with hSP/lCB1R immunoreactivity (Fig. 8*a*,*c*). In contrast, PV-containing neurons were concentrated in the area with lSP/hCB1R immunoreactivity of the cEPN (Fig. 8*b*,*d*). ChAT-containing neurons were few in number and showed nearly uniform localization along the rostrocaudal axis. These results are consistent with the rostrocaudal distribution of EPN neurons in previous studies (Miyamoto and Fukuda, 2015, 2021).

### Four subregions of the EPN based on SP and CB1R immunoreactivities combined with neuronal distributions

**Figure 10.**
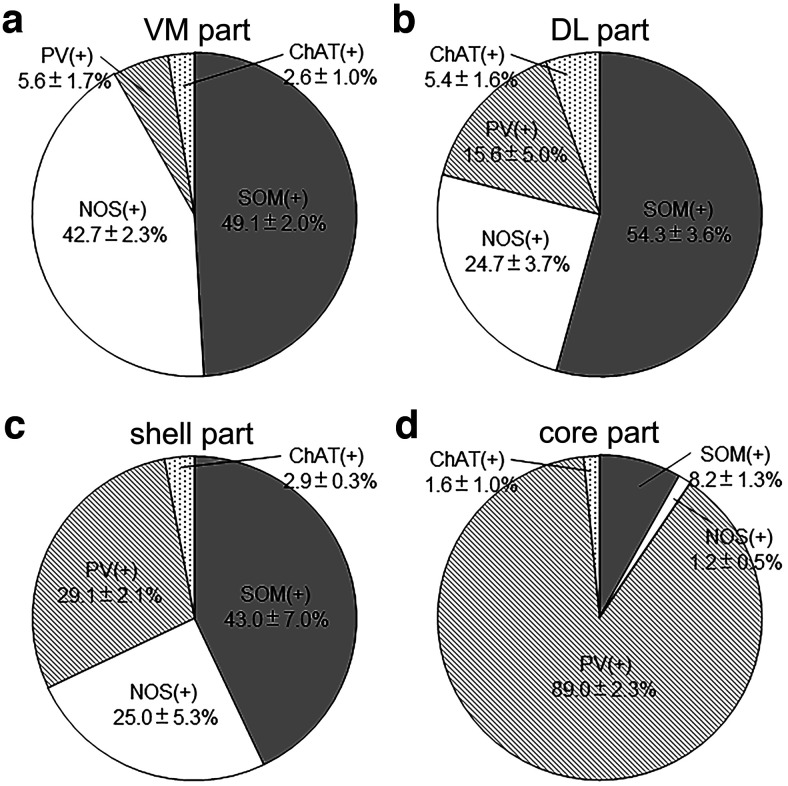
Proportions of four types of EPN neurons in four subregions. ***a–d***, Data are shown as the mean ± SD in the VM (***a***), DL (***b***), shell (***c***), and core (***d***) subregions.

**Figure 11.**
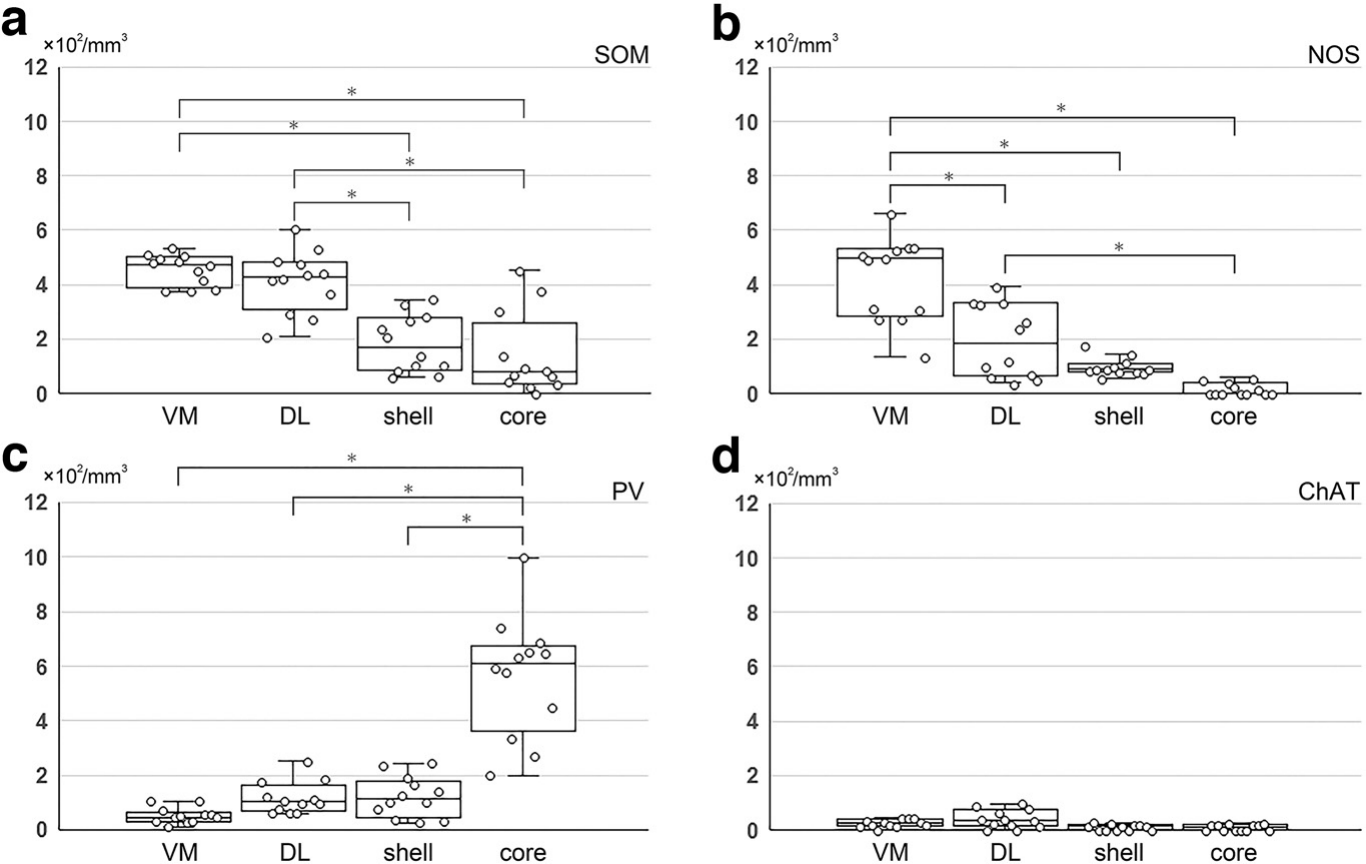
***a–d***, Comparison of the cell density of four types of neurons among different subregions of the EPN. Each circle represents the cell density per unit volume measured in a subregion located in a single section. Measurements in the DL and VM were performed at 4 different positions (−1.04, −1.12, −1.20, and −1.28 mm from bregma) in the rEPN, and those in the core and shell at 4 different positions (−1.36, −1.44, −1.52, and −1.60 mm from bregma) in the cEPN (*n* = 12 sections from 3 animals in each subregion). *p < 0.05, Tukey–Kramer test.

In the above analysis, the EPN was divided into areas with hSP/lCB1R and lSP/hCB1R immunoreactivities. If only these differential immunoreactivities were taken into consideration, the EPN might be classified into two parts. However, the hSP/lCB1R and lSP/hCB1R areas occupied their positions differently in the rEPN and cEPN (Fig. 2). In the rEPN, the hSP/lCB1R-immunoreactive area was located in the VM part, whereas the lSP/hCB1R-immunoreactive area was located in the DL part. In the cEPN, the distribution followed a core and shell pattern in which the lSP/hCB1R-immunoreactive area occupied the core and the hSP/lCB1R-immunoreactive area occupied the surrounding area. Then, a question arises of whether the lSP/hCB1R area in the rostral and caudal EPN should be regarded as one continuous structure, as might be suggested when the red-colored zone in Figure 2 was observed; the same question also concerns the hSP/lCB1R area that might appear in one continuous structure, as seen in the blue-colored zone in Figure 2. Here, we combined the results of the SP and CB1R immunoreactivities with the distribution of four types of EPN neurons, leading to the finding that both lSP/hCB1R and hSP/lCB1R areas could be further divided into their rostral and caudal subregions (Figs. 8, 10, 11). First, although the DL part of the rEPN and the core of the cEPN showed lSP/hCB1R immunoreactivity, the proportions of PV, SOM, and NOS neurons differed greatly between the two subregions (Fig. 10*b*,*d*). Moreover, the cell densities of all SOM, NOS, and PV neurons were significantly different between the DL and core (Fig. 11). Similarly, although the VM and shell shared hSP/lCB1R immunoreactivities, the cell densities of both SOM and NOS neurons were significantly different between the VM and shell (Fig. 11). The cell density of ChAT-containing neurons showed no difference between the parts. These results indicate that both the lSP/hCB1R and hSP/lCB1R areas of the EPN were further classified into the rostral and caudal parts (Fig. 8*c*,*d*) so that the EPN was composed of four subregions as a whole.

Among these subregions, the core in the cEPN was characterized by the highest density of PV neurons (5.73 ± 2.10 × 10^2^/mm^3^) compared with the other three subregions, 0.51 ± 0.29 × 10^2^/mm^3^ in VM, 1.18 ± 0.59 × 10^2^/mm^3^ in DL, 1.02 ± 0.88 × 10^2^/mm^3^ in shell (Fig. 11; *n* = 3 animals; Tukey–Kramer test, *p* < 1.0 × 10^−7^). Similarly, the VM subregion was characterized by the highest density of NOS neurons (4.22 ± 1.54 × 10^2^/mm^3^) compared with the other three subregions: 1.96 ± 1.33 × 10^2^/mm^3^ in DL, 0.79 ± 0.24 × 102/mm^3^ in shell, and 0.04 ± 0.09 × 10^2^/mm^3^ in core (Fig. 11; Tukey–Kramer test, *n* = 3 animals; *p* = 5.2 × 10^−6^ between the VM and DL, *p* < 1.0 × 10^−7^ between the VM and shell, and the VM and core).

### Projection sites of afferent axons from the striatum to the EPN

**Figure 12.**
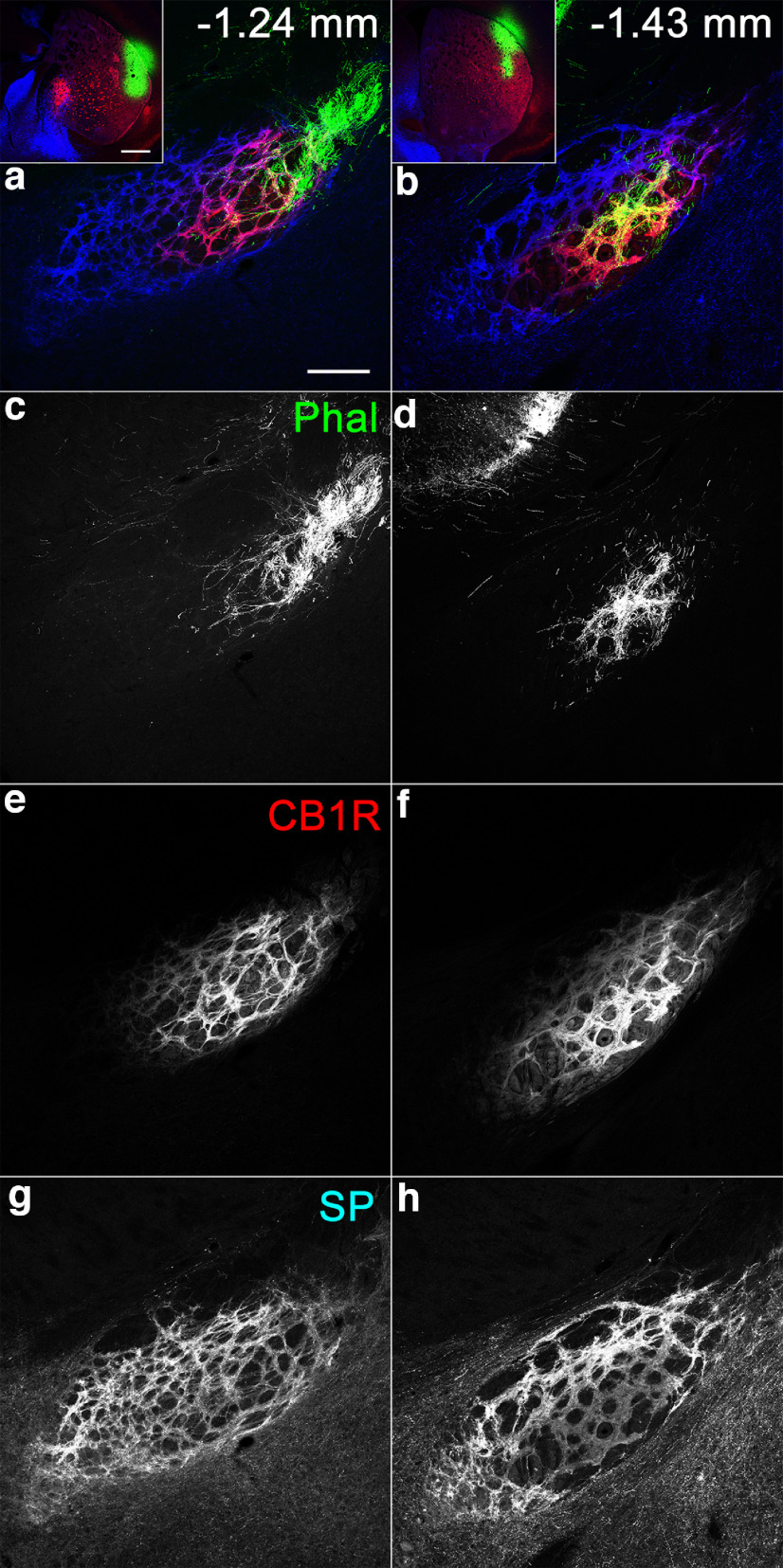
Innervation of areas with lSP/hCB1R immunoreactivity inside the EPN by afferent fibers from the DL part of the striatum, which also shows lSP/hCB1R immunoreactivities. ***a–h***, Pseudocolor images (***a***, ***b***) representing phal (green), CB1R (red), and SP (blue) immunoreactivities are shown separately in ***c*** and ***d***; ***e*** and ***f***; and ***g*** and ***h***, respectively. The sites of phal injection are shown in the insets. The numbers indicate the distance from bregma. Scale bars: ***a***, 200 μm; ***a***, inset,* * 500 μm.

**Figure 13.**
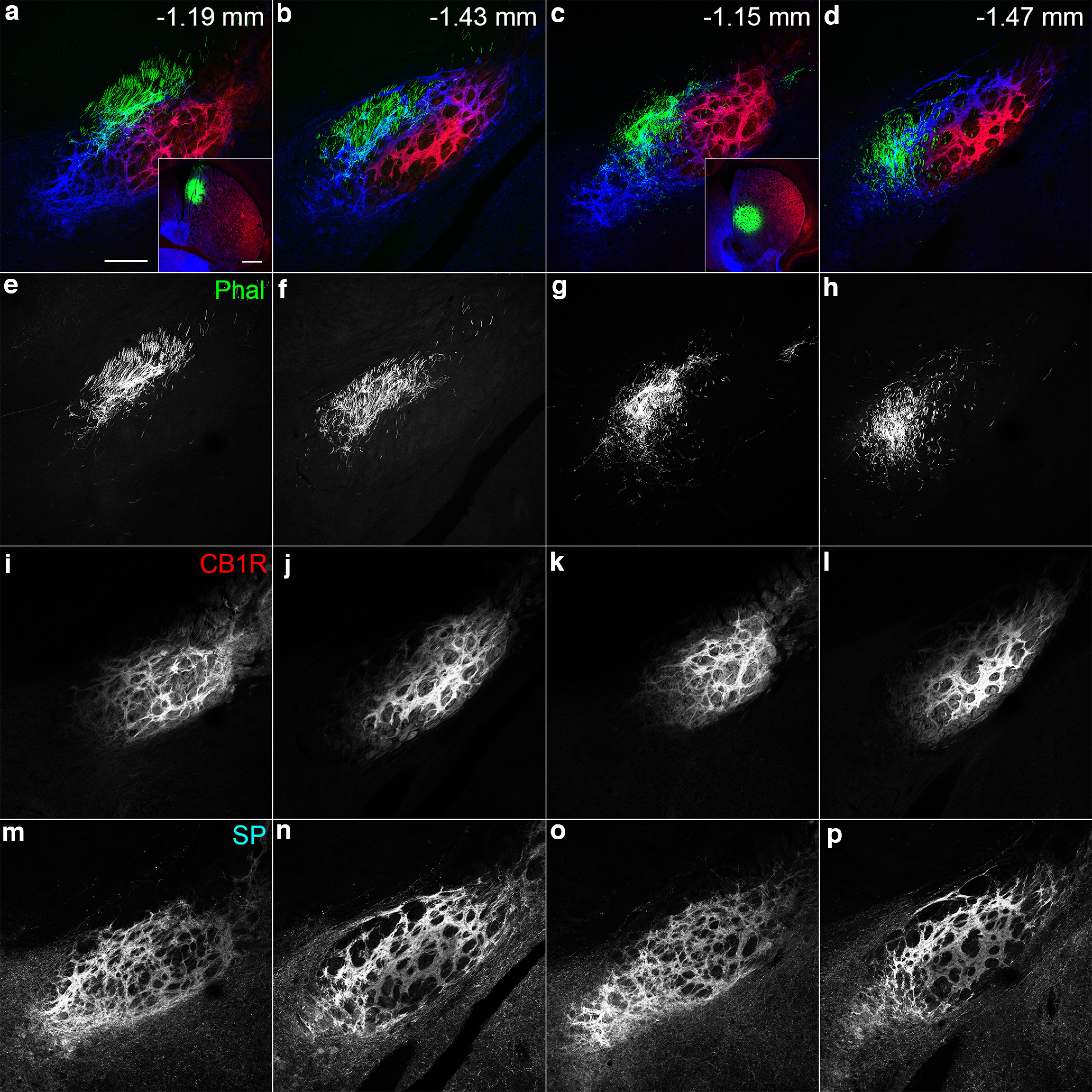
***a***, ***c***, Innervation of areas with hSP/lCB1R immunoreactivity inside the EPN by afferent fibers from the DM (***a***, inset) and VM (***c***, inset) parts of the striatum, which also show hSP/lCB1R immunoreactivities. ***a–p***, Pseudocolor images (***a–d***) representing phal (green), CB1R (red), and SP (blue) immunoreactivities are shown separately in ***e–h***, ***i–l***, and ***m–p***, respectively. The sites of phal injection are shown in the insets. The numbers indicate the distance from bregma. Scale bars: ***a***, 200 μm; ***a***, inset,* *500 μm.

**Figure 14.**
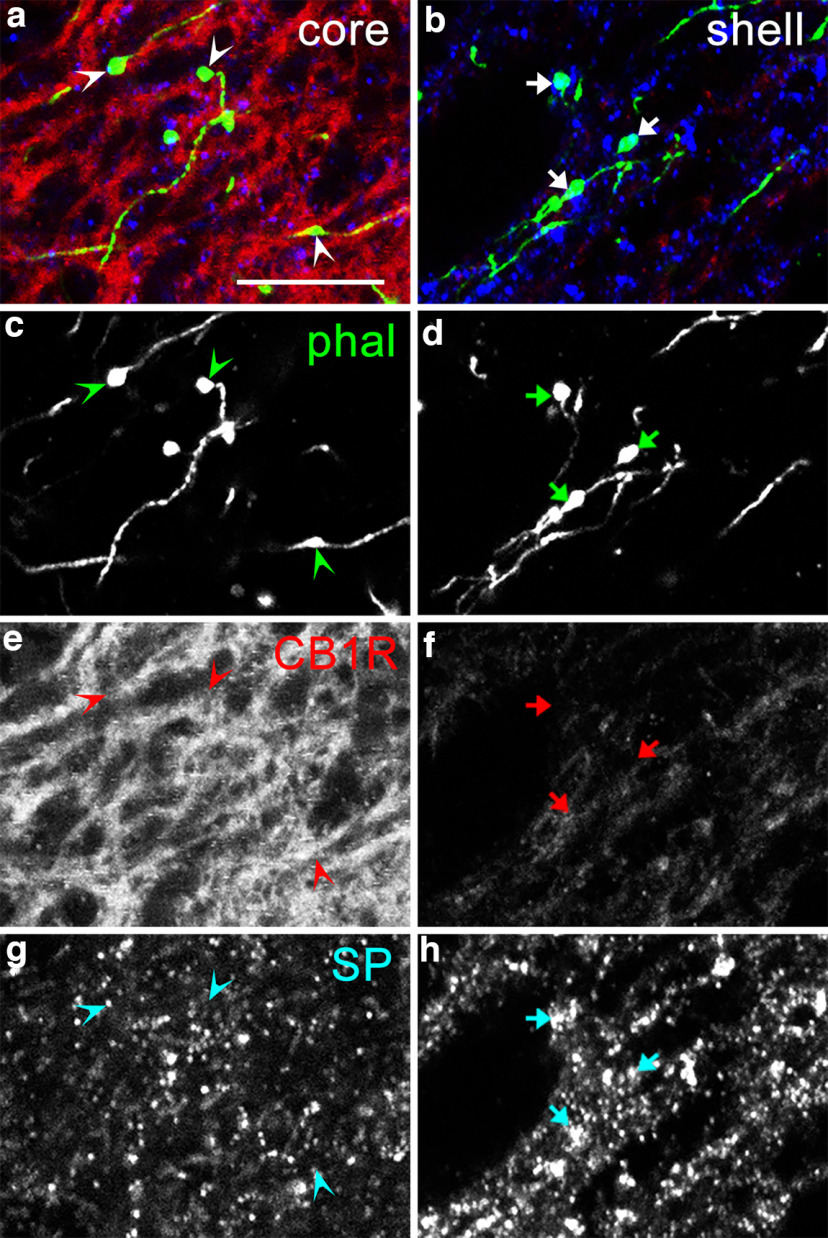
Immunoreactivity of boutons formed along striatum-derived axons in the EPN. ***a***, ***b***, The images in the core (***a***) and shell (***b***) are magnified views of parts of Figures 12*b* and 13*b*, respectively. ***a–h***, Pseudocolor images (***a***, ***b***) representing phal (green), CB1R (red), and SP (blue) immunoreactivities are shown separately in ***c***, and ***d***; ***e***, and ***f***; and ***g*** and ***h***, respectively. Arrowheads and arrows indicate axonal boutons originating from the striatum. CB1R immunoreactivities in phal-labeled boutons in the core are higher than those in the shell, whereas SP immunoreactivities in boutons in the core are lower than those in the shell. Smaller punctate structures in the SP labeling correspond to clusters of SP-positive dense cored vesicles found at the EM level (Shigematsu et al., 2008). Scale bar, 10 μm.

To determine the projection sites of striatoentopeduncular axons in the EPN, anterograde tracer phal was injected into the DL and DM/VM parts of the striatum where lSP/hCB1R and hSP/lCB1R immunoreactivities were observed, respectively. Tracers injected into the DL part of the striatum were predominantly distributed in the DL subregion of the rEPN and core of the cEPN (Fig. 12), both of which corresponded to the lSP/hCB1R-immunoreactive area of the EPN. In contrast, tracers injected into the DM or VM parts of the striatum selectively terminated in the hSP/lCB1R-immunoreactive area of the EPN (Fig. 13), which consisted of the VM subregion of the rEPN and shell of the cEPN. Thus, afferent axons from the striatum to the EPN were projected in a topographic manner with good correspondence in SP/CB1R immunoreactivities. Close observations with higher magnification revealed that characteristic staining pattern in each subregion could be ascribed to the labeling intensity in individual axonal boutons originating from the striatum (Fig. 14).

## Discussion

**Figure 15.**
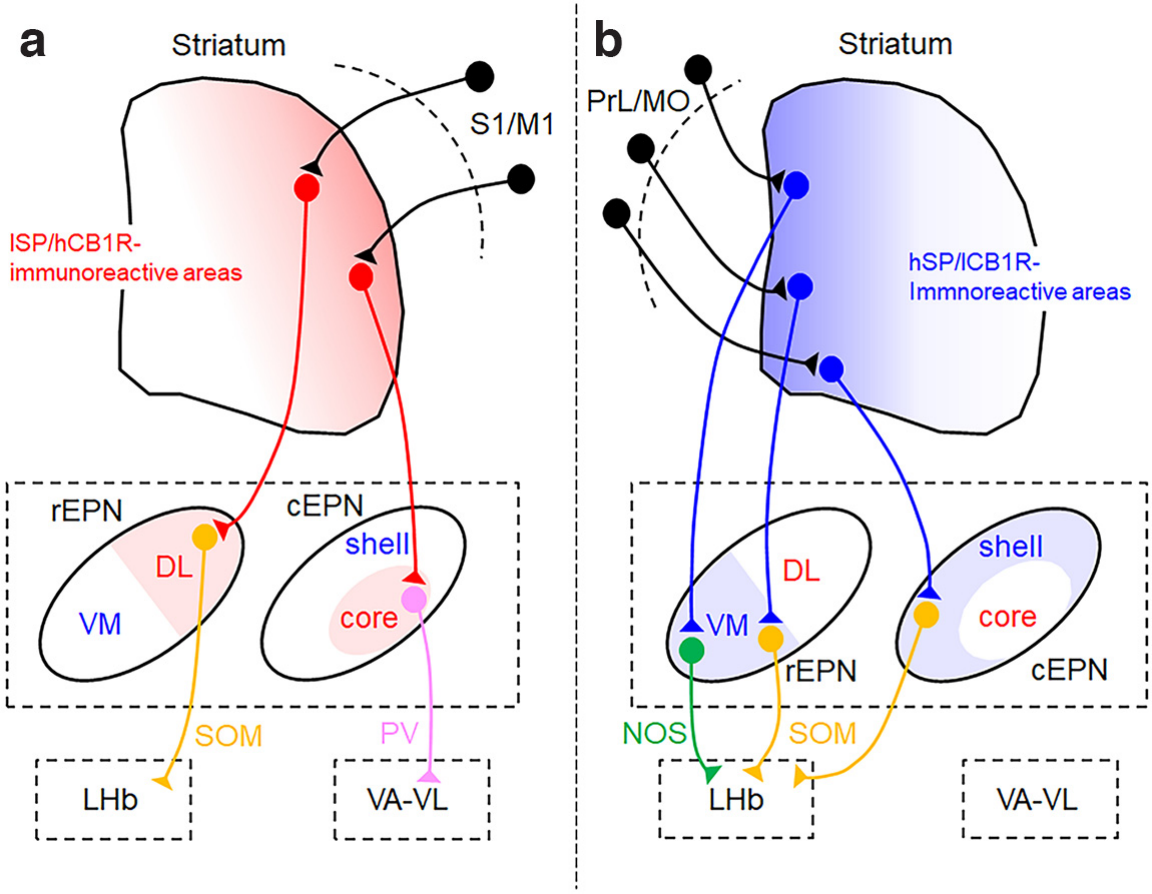
Schematic diagrams of the neural circuit through the basal ganglia, based on both the present results and previous observations (Miyamoto and Fukuda, 2015, 2021; Miyamoto et al., 2018). ***a***, ***b***, The areas shown in red (***a***) and blue (***b***) represent subregions defined by the lSP/hCB1R and hSP/lCB1R immunoreactivities. M1, Primary motor cortex; MO, medial orbital cortex; PrL, prelimbic cortex; S1, primary somatosensory cortex.

In this study, the localization of SP and CB1R immunoreactivities in the EPN was combined with the distribution of four types of neurons to reveal subregions of the EPN. Furthermore, the projection sites of afferent fibers from the striatum were examined in the EPN to demonstrate topographical relationships between the areas of the striatum and EPN that shared similar SP/CB1R immunoreactivities. The present findings are summarized in Figure 15, in which the connectivity already known for each subregion is included.

### Localization of CB1R in the EPN

In this study, we found that CB1R immunoreactivity in the EPN was localized in preterminal axons and axon terminals forming symmetrical synapses. This finding is consistent with previous observations in other nuclei of the basal ganglia, including the striatum, GP, and SNr (Mátyás et al., 2006; Uchigashima et al., 2007). In CLSM images, many GAD-positive boutons showed weak but specific labeling for CB1R, whereas vGluT2-positive boutons did not show CB1R immunoreactivity except for very rare cases. The results of CLSM and EM are consistent with each other and indicate that CB1R in the EPN is localized predominantly in preterminal axons and axon terminals derived from GABAergic neurons, most likely striatal direct pathway neurons. This finding is supported by previous studies showing that the expression of CB1R mRNAs was high in the striatum but was extremely low in the GP and SNr (Matsuda et al., 1990, 1993; Mailleux and Vanderhaeghen, 1992; Hohmann and Herkenham, 2000; Julian et al., 2003). However, the origin of CB1R-expressing axons has been rather controversial. A recent electrophysiological study suggested that blockade of CB1R led to the suppression of long-term depression at STN–EPN synapses (Gorodetski et al., 2018). This finding suggests that CB1R-positive preterminal axons may originate from not only the striatum, but also the STN. Moreover, EM analyses in both previous (Mátyás et al., 2006) and present studies demonstrated that virtually all CB1R-positive axon terminals formed synapses of symmetrical type. Further morphological investigation is required to determine whether there is unambiguous evidence for the localization of CB1R in the STN–EPN projection system.

### Newly defined subregions of the EPN

The complementary immunoreactivities for CBR1 and SP, at first glance, may suggest a dichotomous view that the internal structure of the EPN consists of hSP/lCB1R and lSP/hCB1R regions. However, each of these two regions was further divided according to the compositions of different neuronal types. Thus, the EPN can be divided into the following four subregions: DL and VM subregions in the rEPN, and core and shell subregions in the cEPN.

Each of the four subregions has distinguishing features in morphological aspects (Fig. 15). The core is characterized by the concentration of PV-positive neurons that comprise ∼90% of neurons in this subregion (Fig. 10). Moreover, the core receives inputs from the DL part of the striatum (Fig. 12), where projections from the motor and sensory cortices terminate (Deniau et al., 1996; Ramanathan et al., 2002; Hintiryan et al., 2016; Hunnicutt et al., 2016; Miyamoto et al., 2018). Another important feature is that PV neurons in the core target the ventral anterior–ventral lateral (VA–VL) nucleus of the thalamus (Miyamoto and Fukuda, 2021). Thus, PV neurons correspond to the very neuronal type that had long been considered to represent the canonical circuit of the basal ganglia from the striatum through the EPN toward the VA–VL thalamus, before the recognition of a distinct circuit targeting the lateral habenula (LHb; Matsumoto and Hikosaka, 2007; Hong and Hikosaka, 2008).

A previous study demonstrated that LHb-targeting neurons are distributed throughout the rostral half of the EPN and in the region surrounding the core of the caudal EPN (Miyamoto and Fukuda, 2021). The present study is consistent with and further extends the former analysis of LHb-targeting areas so that the rostral EPN is divided into DL and VM subregions, with the latter containing more NOS neurons, whereas the caudal LHb-targeting area is recognized as a shell subregion (Fig. 15). In a previous study (Miyamoto and Fukuda, 2021), the EPN was simply divided into two parts, core and shell, in which DL and VM subregions of the rEPN defined in the present study were included in the shell. The present results indicate that the term “shell” should be renamed to specify the region located only in the caudal EPN where it surrounds the core.

A recent study using single-cell transcriptional and molecular analysis has uncovered the presence of purely glutamatergic, LHb-targeting PV neurons in the EPN (Wallace et al., 2017). Although PV neurons are concentrated in the core, a small number of PV neurons is located in three other subregions, DL, VM, and shell, just as are SOM neurons (Figs. 8, 11). Because neurons in these subregions send axons to the LHb, sparse PV neurons therein are candidates for LHb-targeting glutamatergic PV neurons. Figure 15 summarizes the present findings that are based on conventional immunohistochemical analysis, and thus it remains to be studied how glutamatergic PV neurons targeting the LHb are incorporated in the scheme.

Heterogeneous SP/CB1R immunoreactivities showed a good correspondence between the striatal subregions and their targets in the EPN; the lSP/hCB1R area in the striatum is connected to the lSP/hCB1R subregions in the EPN, whereas the hSP/lCB1R area in the striatum is connected to the hSP/lCB1R subregions in the EPN (Fig. 15). This finding can be explained at least partially by the presence of extensive local axon collaterals of medium-sized spiny neurons inside the striatum (Wilson and Groves, 1980; Somogyi et al., 1981; Davis et al., 2018); axons and axon terminals showing lSP/hCB1R immunoreactivities provide similar labeling for SP/CB1R in both the striatal collaterals and projection fibers in the EPN subdivisions. Another factor to be considered for high levels of CB1R immunoreactivity in the DL part of the striatum is that axon terminals of cortical afferents in the striatal DL part express CB1R in immunoelectron microscopy (Uchigashima et al., 2007). Therefore, both collateral axon terminals forming inhibitory synapses and cortical axon terminals forming excitatory synapses will explain the high CB1R immunoreactivity in the DL part of the striatum.

We included ChAT neurons in the quantitative analysis of EPN neurons. This was simply because ChAT-positive neurons were located inside the region defined by the boundary of SP immunoreactivity. However, a previous study showed that ChAT-positive neurons were distributed mainly in the GP and its surrounding space and that only a few neurons were found to penetrate into the EPN (Miyamoto and Fukuda, 2015). The present study confirmed this pattern, and their number was found to be small in all of the four subregions (Fig. 8*d*). This corroborates the general idea that ChAT neurons do not belong to the basal ganglia but should be recognized as basal forebrain neurons that are not confined to a restricted area (Zaborszky et al., 2012).

### Functional implications

CB1R localized in presynaptic neurons plays a role in modulating the release of neurotransmitters by the binding of eCB released retrogradely from postsynaptic neurons (Kano et al., 2009). The DSI and DSE produced by this eCB–CB1R binding contribute to synaptic plasticity. Therefore, the presence of two types of axons with high and low CB1R immunoreactivity shown here will represent a functional difference in the strength of synaptic plasticity caused by eCB–CB1R binding. Because the DL subregion of the rEPN and the core subregion of the cEPN are strongly labeled for CB1R, it is assumed that SOM- and PV-containing postsynaptic neurons localized in these areas are powerfully regulated by eCB–CB1R binding to presynaptic axons. The source of these presynaptic axons expressing CB1R in the EPN is the DL part of the striatum (Fig. 15), where cortical afferents from the sensorimotor cortices terminate and also express CB1R on axon terminals (Uchigashima et al., 2007). Thus, it appears reasonable to assume that activities in the sensorimotor loop of the basal ganglia circuit will be strongly influenced by CB1R-induced modulation of corticostriatal and striatoentopeduncular synapses. The present results further suggest sorting of sensorimotor information to the two parallel pathways, one through PV neurons in the core subregion of the cEPN toward the VA–VL thalamus and the other through SOM neurons in the DL subregion of the rEPN toward the LHb; the former will be responsible for smooth motor control, whereas the latter will be responsible for the avoidance of aversive stimuli (Hikosaka, 2010; Li et al., 2019). Alternatively, SOM- and NOS-containing neurons localized in areas with hSP/lCB1R immunoreactivities receive information from the limbic and associative cortices through the medial part of the striatum, target the LHb, and might be less influenced by CB1R-induced plasticity than sensorimotor circuits.

The complementary immunoreactivities for SP and CB1R were first demonstrated in the striatum and SNr (Davis et al., 2018), and we found a similar pattern in the EPN. All of these regions show distinctive labeling patterns for SP/CB1R heterogeneity, on which the structural organization inside each region was elucidated (Davis et al., 2018; this study). However, both SP and CB1R immunoreactivities in the EPN are contained in axons and axon terminals originating from striatal neurons of the direct pathway type. The topographical connectivity examined in the present study was also focused on direct projections from the striatum to the EPN. Importantly, the function of the basal ganglia relies on the coordinated activities of direct and indirect pathways, and the GP targeted by indirect pathway neurons is also labeled for CB1R. Therefore, how the outcomes of future analyses regarding the indirect pathway can be incorporated into the results obtained in the present study requires further research.

## Synthesis

Reviewing Editor: Katalin Toth, University of Ottawa Decisions are customarily a result of the Reviewing Editor and the peer reviewers coming together and discussing their recommendations until a consensus is reached. When revisions are invited, a fact-based synthesis statement explaining their decision and outlining what is needed to prepare a revision will be listed below. The following reviewer(s) agreed to reveal their identity: Ferenc Matyas.

This is an interesting and expertly conducted study with high quality data and figures. Both reviewers are positive, but they both raised several questions that the authors would need to address, the most important questions are related to the antibodies used and the number of neuronal groups that have been identified.

rev.#1.

The Authors of this MS proposed that mouse entopeduncular nucleus (EPN) can be divided into subregions based on immunostaining using antibodies developed against SP and CB1. Overall, the analysis and the quality of the experiments are convincing. I have the following comments to this MS.

i) Recently, it has been uncovered that in addition to the thalamus-projecting inhibitory PV neurons in the mouse EPN there are glutamatergic PV neurons that project to the LHb (Wallace et al., 2017 Neuron), a cell population which is not mentioned in this work. Could the Authors fit this cell type into their circuit diagram? It would be interesting to see where these glutamatergic PV neurons locate within the EPN.

ii) The antibody used in this study to visualize CB1 reveals these receptors on GABAergic axon endings, but not on excitatory boutons. Therefore, it is misleading to suggest that Vglut2-containing axon terminals lack CB1.

iii) It would be very elegant to directly show that striatal efferents express CB1 or SP in the EPN using multi-channel confocal microscopy. Thus, CB1 or SP immunoreactivity of Phal-labelled fibers in the EPN would be ideal to demonstrate.

iv) The Authors sum up the number of PV, SOM, NOS and Chat neurons to examine the proportion of these cells. Do the Author have evidence that there are no further cell types in the mouse EPN? Have they performed e.g. a NeuN staining to demonstrate that there are no further, unlabeled neurons in the EPN?

v) Related to this later question: In humans and primates no Chat neurons were reported to be present in the globus pallidus internus, a homologous to the mouse EPN. In mice having a small brain the EPN is adjacent to the nucleus basalis where Chat neurons are abundant. Previous studies have already proposed that in rodents Chat neurons do not confine to a very restricted area, since nucleus basalis is hard to define, but they can be present in neighboring regions, including the EPN (Gielow & Zaborszky, 2017). Thus, based on these thoughts, Chat neurons in the EPN are not part of the basal ganglia, but belong to the basal forebrain. In line with this suggestion the Authors found a diffuse distribution of Chat neurons in the EPN. I would encourage the Authors to discuss this point if they agree.

vi) In the methods the Authors stated that anti-Calb antibody was purchased from Swant (l 138), yet a different antibody is listed in the Table. Can the Authors double-check the list of antibodies?

Rev. #2.

The entopeduncular nucleus is considered to be one of the major output nuclei of the basal ganglia. Still, little is known about its architecture as well as information process occurred within its territory. To make a step forward to fulfill these gaps, the present study investigates the internal structures of EPN which are necessary for any further anatomical and functional investigations. The authors found that striatal inputs, expressing either CB1 or SP segregate EPN into two major regions, which could be further divided into 2-2 additional subregions based on the distribution of different neuronal elements. Furthermore, this parcellation follows the topographic striatal inputs shown by anterograde tracing.

This is a well-conducted study with beautiful presentation.

I do not have fundamental criticism; still, there are some observations that are worth to be considered.

1) The authors used 4 different neuronal types to subdivide the EPN. Based on this, 4 distinct regions have been defined. This is only valid if these 4 groups of neurons cover the entire neuronal population of EPN. If there is (are) additional cell type(s) beside PV, SOM, Chat and NOS, further subregion(s) could be identified.

So, the question arises: what proportion of the EPN neurons can be labelled with Chat, SOM, PV and NOS? Do these 4 markers cover the entire neuronal elements in the EPN?

2) There are many antibodies against CB1 in the market, which can label CB1-profiles with different intensities. For example, CB1 immunostaining in the central amygdala can be varying using distinct CB1 antibody. The present study show no (or very weak) signal in the CeA, but Ramikie et al., 2014 presented moderate level of CB1 expression. The question is whether the CB1 immunostaining in the EPN could show similar differences with distinct antibodies or not.

Minors.

a) Rodent brain atlases and studies use the ‘medial globus pallidus’ terminology for EPN regions in addition to the internal globus pallidus. It should be mentioned for clarity.

b) Although it is stated that most of the CB1 profiles correspond to axonal elements, ISH data shows CB1 mRNA in the EPN. Have the authors found any somatodendritic labelling using either confocal or electron microscopy? If yes, which neuronal types express CB1 in the EPN?

c) Fig 8c-d, the midpoint seems to be arbitrary. Based on the distribution of neuronal subtypes, the borderline could be drawn around -1.44.

d) Fig 14. Indicating the reference for the previous observations in the legend could be really helpful for the readers.
